# Safety and Efficacy of Achieving Very Low LDL Cholesterol Concentrations with PCSK9 Inhibitors

**DOI:** 10.3390/jcm14134562

**Published:** 2025-06-27

**Authors:** Akshay Machanahalli Balakrishna, Sharanya Kaushik, Sangeetha Tandalam Palanivelu, Noorhan Monther, Shiva P. Ponamgi, Venkata Mahesh Alla, Shantanu M. Patil

**Affiliations:** 1 Division of Cardiovascular Medicine, Department of Medicine, Creighton University School of Medicine, Omaha, NE 68124, USA; 2 Department of Internal Medicine, Jacobi Medical Center, NYC H+H, Albert Einstein College of Medicine, New York, NY 10461, USA; 3 Department of Internal Medicine, Creighton University School of Medicine, Omaha, NE 68124, USA

**Keywords:** PCSK9 inhibitors, very low LDL, major adverse cardiovascular events, adverse events

## Abstract

**Background:** The advent of newer pharmacological agents, particularly proprotein convertase subtilisin/kexin type 9 (PCSK-9) inhibitors, in combination with conventional lipid-lowering treatments, has allowed for the significant lowering of low-density lipoprotein cholesterol (LDL-C). However, it is unclear if very low LDL-C levels achieved with the use of PCSK-9 inhibitors are associated with increased adverse events that may outweigh potential benefits. **Methods:** A systematic search of PubMed, Medline, and Cochrane databases was conducted from their inception to 21 February 2025, for randomized controlled trials (RCTs) reporting clinical outcomes with intensive lipid-lowering treatment with PCSK-9 inhibitors leading to very low (<40 mg/dL) LDL-C levels vs. a control group with higher LDL-C levels. The outcomes of interest included the incidence of major adverse cardiovascular events (MACEs), neurocognitive disorders, diabetes mellitus, muscle disorders, any adverse events, events leading to drug discontinuation, cataract, hepatobiliary disorders, and cancer. Random effects meta-analysis models were used to calculate the pooled incidence and odds ratio (OR) with 95% confidence intervals (Cis). **Results:** A total of six RCTs with 52,951 patients (11,209 very low LDL-C, and 41,742 control) met the inclusion criteria. Compared with patients in the control arm, very low LDL-C was associated with a reduction in MACEs (OR = 0.76, 95% CI: 0.64, 0.89; *p* < 0.01; I^2^ = 44.8%). The incidence of most safety outcomes including neurocognitive disorders, diabetes mellitus, muscle disorders, any adverse events, events leading to drug discontinuation, cataract, hepatobiliary disorders, and cancer were comparable between the very low LDL-C and control groups. **Conclusions:** Very low LDL-C values following intensive lipid-lowering with PCSK-9 inhibitors are associated with a major reduction in cardiovascular events without any significant increase in serious side effects.

## 1. Introduction

Epidemiological studies have abundantly demonstrated that LDL-cholesterol (LDL-C) is a modifiable risk factor for atherosclerotic cardiovascular disease, and lowering LDL-C levels has been consistently associated with improved cardiovascular outcomes [1,2,3,4]. The introduction of statins revolutionized cardiovascular prevention by significantly lowering LDL-C and reducing major adverse cardiovascular events (MACEs) [2,3]. However, despite optimal statin therapy, many high-risk patients fail to achieve guideline-recommended LDL-C targets, leaving residual cardiovascular risk [5,6,7]. This therapeutic gap has spurred the development of novel lipid-lowering agents, most notably proprotein convertase subtilisin/kexin type 9 (PCSK-9) inhibitors, which have enabled unprecedented reductions in LDL-C [1]. PCSK-9 inhibitors, such as evolocumab and alirocumab, are monoclonal antibodies that bind circulating PCSK-9, preventing the degradation of hepatic LDL receptors and thereby enhancing LDL-C clearance. When added to statin therapy, these agents can lower LDL-C by an additional 50–60%, with many patients achieving levels well below conventional targets [8,9,10].

Current societal guidelines recommend an LDL-C reduction of ≥50% from baseline for patients at very high cardiovascular risk, with a target of <55 mg/dL. In cases of atherosclerotic cardiovascular disease where a second vascular event occurs within two years despite maximal statin therapy, an even more aggressive target of <40 mg/dL may be considered. For primary prevention, high-risk individuals are advised to aim for <70 mg/dL, while moderate- and low-risk patients target < 100 mg/dL [11,12]. The extent to which LDL-C levels can be safely decreased is unclear, even though the clinical benefit of lowering LDL-C is evident across the entire spectrum of baseline LDL-C. Apprehension regarding possible adverse effects associated with very low LDL-C, such as hemorrhagic stroke and diabetes mellitus, has risen due to small observational studies, leading to the discontinuation of drugs in high-risk individuals [13,14]. To address this gap, we conducted a meta-analysis of randomized controlled trials (RCTs) to evaluate the safety and clinical benefits of LDL-C reduction to very low levels with PCSK-9 inhibitors.

## 2. Methods

### 2.1. Data Sources and Search Strategy

A systematic review of published articles was conducted following the PRISMA (Preferred Reporting Items for Systematic Reviews and Meta-Analyses) guidelines [15]. A comprehensive search of MEDLINE, EMBASE, and the Cochrane Library was conducted from inception to 5 January 2025, using relevant medical subject headings and variations. The following search terms and keywords were used: ‘PCSK-9 inhibitor’, ‘very low LDL’, ‘safety’, ‘adverse events’, ‘major adverse cardiovascular events.’ Two independent researchers (A.M.B. and S.K.) conducted the initial screening process, meticulously examining each study’s title and abstract to assess eligibility. When records appeared to meet preliminary inclusion criteria, the reviewers obtained and carefully evaluated full-text articles against the predefined selection parameters. To ensure efficient reference management, all identified studies were imported into EndNote X9 (Clarivate Analytics, Philadelphia, PA, USA), where an initial deduplication process was performed using the software’s automated detection features supplemented by manual verification.

During the screening phase, any disagreements between reviewers regarding study inclusion were systematically documented and subsequently resolved through consensus discussions with the senior investigator (S.M.P.), who provided final arbitration when needed. This rigorous dual-review approach was implemented to minimize selection bias and enhance the reliability of study inclusion decisions. Regarding ethical considerations, since this analysis exclusively utilized previously published, de-identified data from publicly available sources, a formal ethics committee review was deemed unnecessary according to current institutional policies governing secondary data analysis.

### 2.2. Study Selection

Inclusion Criteria: Eligible studies were randomized controlled trials (RCTs) that evaluated PCSK-9 inhibitor therapy, achieving ultra-low LDL-C levels (defined as <40 mg/dL), and compared them against control groups with LDL-C ≥ 40 mg/dL. Additionally, studies had to have a minimum follow-up period of six months and report at least one relevant clinical outcome.

Exclusion Criteria: Studies were excluded if they did not involve PCSK-9 inhibitors, lacked the specified outcomes, or were non-human studies, conference abstracts, or unpublished reports.

Rationale for LDL-C Threshold: The 40 mg/dL cutoff for defining very low LDL-C aligns with the most stringent target recommended by the European Society of Cardiology for patients at extreme cardiovascular risk, as outlined in their dyslipidemia guidelines [11].

Risk of Bias Assessment: The methodological quality was evaluated using the Cochrane Collaboration’s risk of bias tool (see Supplementary Figure S1).

### 2.3. Outcomes

The outcomes of interest included the incidence of MACEs, neurocognitive disorders, diabetes mellitus, muscle disorders, any adverse events, events leading to drug discontinuation, cataract, hepatobiliary disorders, and cancer. MACEs were defined by a composite of cardiovascular death, myocardial infarction, and stroke. All outcomes were classified according to the definition used in each investigation, and the definitions were similar across all the included trials.

### 2.4. Statistical Analysis

To evaluate the heterogeneity among the included studies, the Higgins I^2^ (I-squared) statistic was employed, a widely accepted measure in meta-analyses that quantifies the degree of inconsistency across study results. An I^2^ value of 0% signifies complete homogeneity, indicating that any observed variability is due to chance alone, whereas progressively higher values suggest increasing heterogeneity attributable to genuine differences in effect sizes. For practical interpretation, conventional benchmarks were applied, classifying I^2^ values of 25%, 50%, and 75% as indicative of low, moderate, and high heterogeneity, respectively. These thresholds help contextualize the potential impact of variability on the meta-analysis conclusions.

To assess potential publication bias, which could skew the pooled results if smaller or non-significant studies were systematically omitted, funnel plots were generated and visually inspected for asymmetry. This approach was restricted to outcomes with data from at least three studies, as fewer studies would render the funnel plot unreliable. For dichotomous outcomes, odds ratios (ORs) accompanied by 95% confidence intervals (CIs) were computed to summarize the treatment effects reported in each study. These study-specific estimates were then synthesized using the DerSimonian and Laird random-effects model, chosen for its ability to account for between-study variance by incorporating heterogeneity estimates derived from the Mantel–Haenszel method. This conservative approach ensures broader, more generalizable CIs when heterogeneity is present.

Statistical significance was determined using a two-tailed alpha level of *p* ≤ 0.05, with the overall effect size further validated by a corresponding z-value, reinforcing inferences drawn from the 95% CIs. All statistical procedures were executed using Cochrane Review Manager (RevMan) version 5.3 (The Nordic Cochrane Center, The Cochrane Collaboration, Copenhagen, Denmark), a specialized software package designed for systematic reviews and meta-analyses. To uphold transparency and methodological rigor, the findings were reported following the Preferred Reporting Items for Systematic Reviews and Meta-Analyses (PRISMA) 2020 guidelines [15]. Additionally, a supplementary PRISMA 2020 checklist was compiled to ensure compliance with the updated reporting standards, further enhancing the reproducibility and reliability of the analysis.

## 3. Results

### 3.1. Search Results

**Figure 1:**
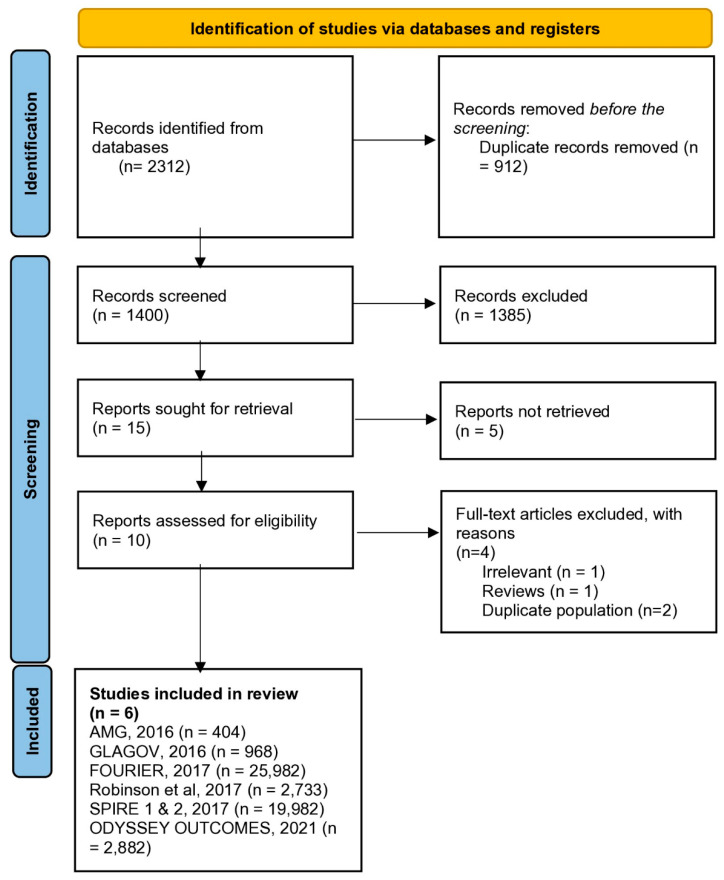
Preferred Reporting Items for Systematic Reviews and Meta-Analyses flow diagram of study selection.

Six RCTs [16,17,18,19,20,21], including 52,951 patients (11,209 very low LDL-C and 41,742 control), were selected and included in the meta-analysis (Figure 1). Table 1 presents the characteristics of all the included studies.

Demographic and clinical characteristics were well-balanced between the very low LDL-C cohort and control group. Both populations had identical mean ages of 61 years, with comparable gender distributions (28% versus 32% female participants). The prevalence of major comorbidities showed minimal variation between groups: hypertension affected approximately 79% of patients in both cohorts, while diabetes mellitus was present in 37% of the intervention group versus 35% of controls. Smoking status was similarly distributed, with current smokers comprising 28% and 25% of the very low LDL-C and control groups, respectively. Complete baseline demographic and clinical data are comprehensively detailed in Table 2. The study duration averaged 1.7 years of follow-up time (median value).

Methodological quality assessment was performed using the validated Cochrane Risk of Bias Tool [22], which systematically evaluates potential sources of bias across multiple domains. Our analysis revealed no substantial methodological concerns across the included studies, with detailed quality assessment data provided in the Supplementary Materials. Publication bias evaluation through a funnel plot analysis indicated symmetrical distribution patterns for all measured outcomes, suggesting a minimal likelihood of reporting bias influencing our findings.

### 3.2. Safety Outcomes

**Figure 2:**
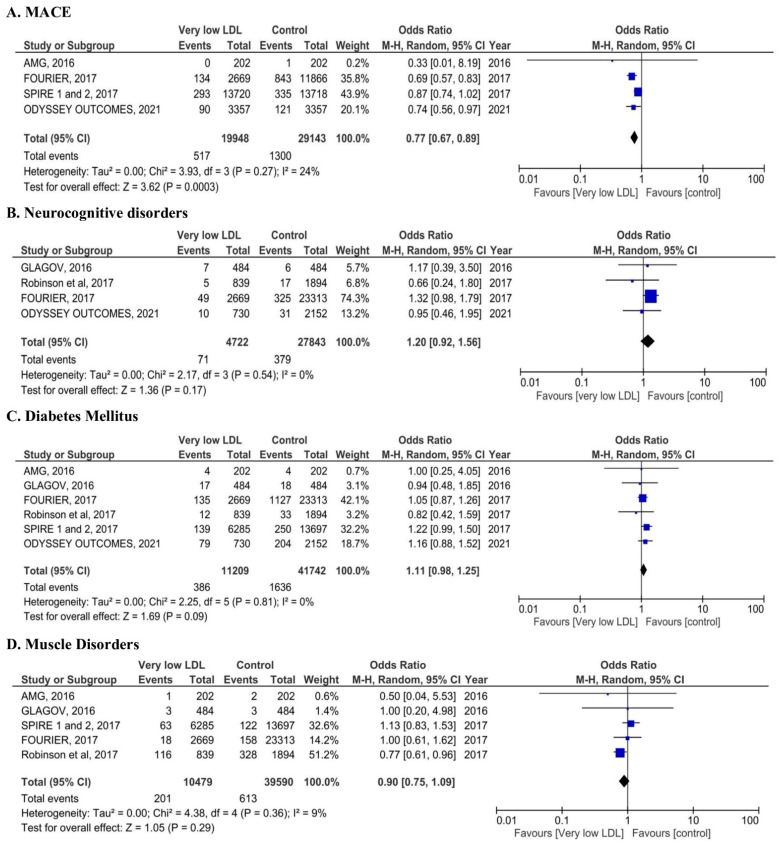

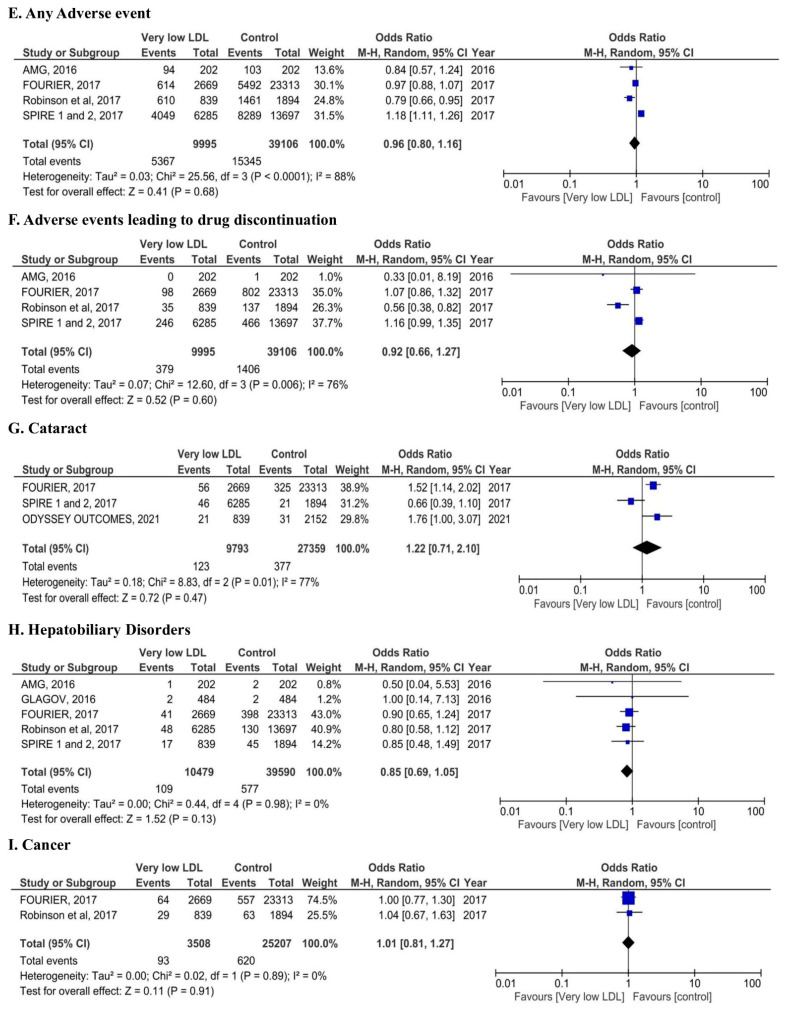
Forest plot of studies with odds ratios for (**A**) MACEs; (**B**): neurocognitive disorders; (**C**): diabetes mellitus; (**D**): muscle disorders in the very low low-density lipoprotein cholesterol group vs. control group. Forest plot of studies with odds ratios for (**E**): any adverse events; (**F**): adverse events leading to drug discontinuation; (**G**): cataract; (**H**): hepatobiliary, (**I**): cancer in the very low low-density lipoprotein cholesterol group vs. control group. Legend: Odds ratio for individual studies are indicated by squares and 95% CIs by horizontal lines. Overall totals and their 95% CIs are represented by diamonds, in which the diamond’s center denotes the point estimate, and the width denotes the 95% CI. The size of the squares and the diamonds are proportional to the statistical information conveyed.

Compared with patients in the control arm, very low LDL-C was associated with a reduction in MACEs (OR = 0.77, 95% CI: 0.67, 0.89; *p* < 0.01; I^2^ = 24%, Figure 2A). The incidence of other safety outcomes including neurocognitive disorders (OR = 1.20, 95% CI: 0.92, 1.56; *p* = 0.17; I^2^ = 0%, Figure 2B), diabetes mellitus (OR = 1.11, 95% CI: 0.98, 1.25; *p* = 0.09; I^2^ = 0%, Figure 2C), muscle disorders (OR = 0.90, 95% CI: 0.75, 1.09; *p* = 0.29; I^2^ = 9%, Figure 2D), any adverse events (OR = 0.96, 95% CI: 0.80, 1.16; *p* = 0.68; I^2^ = 88%, Figure 2E) were comparable between the very low LDL-C and control groups. Importantly, adverse events leading to drug discontinuation (OR = 0.92, 95% CI: 0.66, 1.27; *p* = 0.6; I^2^ = 76%, Figure 2F) did not show any statistical difference between the very low LDL-C and control groups. Additionally, odds of other events such as cataract (OR = 1.22, 95% CI: 0.71, 2.1; *p* = 0.47; I^2^ = 77%, Figure 2G), hepatobiliary disorders (OR = 0.85, 95% CI: 0.69, 1.05; *p* = 0.13; I^2^ = 0%, Figure 2H), and cancer (OR = 1.01, 95% CI: 0.81, 1.27; *p* = 0.91; I^2^ = 0%, Figure 2I) were comparable between the very low LDL-C and control groups. No publication bias was found in any of the analyses mentioned above.

## 4. Discussion

Our meta-analysis of six RCTs found that the very low LDL-C group experienced significantly lower rates of MACEs. In addition, the incidence of safety adverse outcomes, including neurocognitive disorders, diabetes mellitus, muscle disorders, any adverse events, events leading to drug discontinuation, cataract, hepatobiliary disorders, and cancer, was similar between the very low LDL-C and control groups.

### 4.1. Cardiovascular Benefits of Very Low LDL-C

The pooled analysis of MACEs demonstrated a significant 23% reduction in risk, highlighting the strong cardiovascular benefit of intensive LDL-C lowering. This aligns with the results of prior trials, including FOURIER and ODYSSEY OUTCOMES, which demonstrated substantial reductions in the risk of MACEs in the very low LDL-C group [18,21]. Our results extend the findings of prior studies showing that each 1 mmol/L (38.7 mg/dL) reduction in LDL-C decreases cardiovascular events by 22% [23]. Prior research has shown that very low LDL-C levels effectively reduce cardiovascular complications without major safety concerns, although the ability to detect rare events in these trials has been limited by the smaller study size [24]. Furthermore, the lower number of MACEs can be explained by progressively higher regression of coronary atherosclerotic plaque with lower LDL-C levels that also seems to be valid even at extremely low LDL-C levels of 7 mg/dL without a plateauing effect [25]. This was also explained in the GLAGOV trial, where evolocumab effectively reduced coronary plaque volume (intravascular ultrasound-measured percent atheroma volume reduction of 0.95% vs. placebo, *p* = 0.001) and provided cardiovascular benefits even at LDL-C levels as low as 20 mg/dL, well below current targets [17]. This supports the biological plausibility of our findings, as plaque stabilization and regression likely mediate the clinical benefits observed. PCSK9 inhibitors promote plaque stabilization not only by increasing fibrous cap thickness but also by reducing lipid-rich necrotic core volume, as demonstrated in the PACMAN-AMI trial [26]. Unlike statins, which may increase plaque calcification, PCSK9 inhibitors appear to modulate plaque composition without altering calcification patterns [27]. While gray-scale median data are sparse, intravascular ultrasound studies consistently show the regression of percent atheroma volume, supporting their role in reversing atherosclerosis [26,27]. Recent evidence suggests there may be no lower threshold for LDL-C benefit. The HUYGENS study showed that achieving LDL-C levels < 20 mg/dL with evolocumab resulted in greater coronary plaque stabilization (increase in minimum fibrous cap thickness by 42.7 μm vs. 21.5 μm with placebo, *p* = 0.01) [27]. These imaging findings correlate with our clinical outcomes, suggesting that “lower is better” remains valid even at extremely low LDL-C levels.

### 4.2. Safety Profile of Intensive LDL-C Lowering

#### 4.2.1. Neurocognitive Effects

The lack of association between very low LDL-C and neurocognitive disorders (OR 1.20, 95% CI 0.92–1.56) is supported by multiple lines of evidence. Mendelian randomization studies of individuals with lifelong low LDL-C due to PCSK9 and hydroxy-3-methylglutaryl-CoA reductase variants show no increased dementia risk [28]. Our study, along with other large observational studies, has consistently shown that people with genetically low LDL-C levels do not have an elevated risk of neurocognitive diseases such as Parkinson’s disease, vascular dementia, and Alzheimer’s disease [28,29]. The EBBINGHAUS study further supports the safety of lowering LDL-C with non-statin medications such as PCSK9 inhibitors added to statin therapy, which evaluated the effect of very low LDL-C on cognitive performance [30]. In the EBBINGHAUS study, more than 50% of the study population (661/1204 patients) achieved an LDL-C level of less than 25 mg/dL with no change in cognition using the Cambridge Neuropsychological Test [30].

#### 4.2.2. Diabetes Risk

While previous trials have linked intensive statin therapy with an increased risk of diabetes [31], our meta-analyses did not show any significant increased risk of diabetes after lowering LDL-C significantly with PCSK9 inhibitors. Due to the paucity of individual clinical data, it is hard to confirm if this is due to patient characteristics such as the prevalence of prediabetes or if this is related to the class of medications, i.e., statins vs. non-statins [32]. Unlike statins, which increase diabetes risk by 9–12% [31], our analysis found no significant association between PCSK9 inhibitors and new-onset diabetes (OR 1.11, 95% CI 0.98–1.25). This is biologically plausible, as PCSK9 inhibitors do not impair glucose metabolism like statins [33]. The FOURIER trial specifically analyzed HbA1c changes and found no difference between evolocumab and placebo groups (0.05% vs. 0.04%, *p* = 0.60) [8,34]. However, a longer-term follow-up is needed to confirm this safety profile. However, this provides reassurance that the risk of diabetes is not directly due to LDL-C lowering or PCSK9 inhibition [14,35,36].

#### 4.2.3. Myopathy and Cataract

In our meta-analyses, we found no significant difference in the overall incidence of cataract after lowering LDL-C to very low levels. The absence of increased muscle symptoms is particularly relevant for statin-intolerant patients. This aligns with the GAUSS-3 trial, where evolocumab caused significantly fewer muscle symptoms than atorvastatin (20.7% vs. 28.8%, *p* = 0.006) [37]. The mechanism may involve the avoidance of statin-induced mitochondrial dysfunction [38]. Prior studies have shown that the true incidence of myopathy is very low with statin use, but it still leads to a high risk of drug discontinuation [39,40]. As seen in previous studies, this may be to a due to a negative placebo (nocebo effect), myalgia without myopathy, and disinformation present in social media [41]. Our study highlights that LDL-C can be lowered safely with PCSK9 inhibitors without any adverse muscle effects. The incidence of cataract after achieving very low LDL-C has been inconsistent. FOURIER and ODYSSEY OUTCOME suggest a potential increase in cataract risk [18,21], while SPIRE 1 and 2 had contrasting results with a possible decreased risk of cataracts [20].

#### 4.2.4. Clinical Standpoint

The absence of increased MACEs and drug discontinuation rates due to any adverse events is particularly reassuring. These endpoints carry the most significant weight when considering the long-term adoption of intensive LDL-C lowering strategies. While other adverse events such as cataracts or minor muscle symptoms are important to monitor, they do not carry the same clinical implications as MACEs or treatment discontinuation. The data suggest that despite profound LDL-C lowering, patients are not at higher risk of critical adverse outcomes that would necessitate the cessation of therapy or lead to major cardiovascular harm [26,38]. This supports the safety and tolerability of PCSK9 inhibitors in achieving very low LDL-C levels.

#### 4.2.5. Mechanistic Considerations

The safety of very low LDL-C with PCSK9 inhibitors may relate to their mechanism of action. Unlike statins, which reduce intrahepatic cholesterol synthesis, PCSK9 inhibitors enhance LDL receptor recycling without affecting intracellular cholesterol pools [42]. This may preserve steroidogenesis and cell membrane integrity while still achieving profound LDL-C reduction.

Emerging data suggest PCSK9 inhibitors may have pleiotropic benefits beyond LDL-C lowering, including reduced lipoprotein (a) levels by 25–30%, anti-inflammatory effects (reduced high-sensitivity C-reactive protein), improved endothelial function [43,44,45]. These effects may contribute to the cardiovascular benefits observed in our analysis.

#### 4.2.6. Clinical Implications

Our findings support more aggressive LDL-C targets in high-risk patients for secondary prevention in patients with progressive ASCVD, and despite statin therapy, an LDL-C target < 40 mg/dL appears safe and effective. For primary prevention in very high-risk patients (e.g., familial hypercholesterolemia), targets < 55 mg/dL are justified PCSK9 inhibitors and should be considered earlier in the treatment algorithm for high-risk patients, not just as a last-line therapy. The 2022 ACC Expert Consensus Pathway on Novel Therapies for LDL-C Lowering now recommends considering PCSK9 inhibitors as first-line therapy for certain very high-risk patients [46]. Our safety data support this paradigm shift.

#### 4.2.7. Economic Considerations

While not addressed in our analysis, cost-effectiveness remains a barrier to widespread PCSK9 inhibitor use. However, recent price reductions and outcome-based contracts have improved affordability [47]. The FOURIER analysis found evolocumab became cost-effective at USD 9669/year when LDL-C remained ≥100 mg/dL on statins [48]. As generic versions emerge, utilization will likely increase. Annual costs for PCSK9 inhibitors (e.g., evolocumab: approximately USD 5800; alirocumab: approximately USD 5400) far exceed those of statins (less than USD 100/year) or ezetimibe (approximately USD 300/year). However, cost-effectiveness analyses from the FOURIER and ODYSSEY OUTCOMES trials suggest that PCSK9 inhibitors may become economically viable in high-risk populations (e.g., secondary prevention with baseline LDL-C ≥ 100 mg/dL despite statins), with incremental cost-effectiveness ratios approaching USD 50,000–100,000 per quality-adjusted life-year after price reductions [47,48].

Real-world uptake is further hindered by prior authorization requirements and restrictive insurance formularies. Many patients do not receive PCSK9 inhibitors due to administrative hurdles, despite guideline recommendations. In contrast, generic statins and ezetimibe face no such barriers, underscoring disparities in access [49].

Emerging strategies such as outcome-based contracts and biosimilar competition may improve affordability. Policymakers must balance these innovations against the long-term societal cost of untreated hyperlipidemia. Future economic models should incorporate the potential cost offsets from reduced hospitalizations and revascularizations, as observed in the GLAGOV trial [17].

#### 4.2.8. Alternative Therapies

While PCSK9 inhibitors offer profound LDL-C reduction, alternative therapies like ezetimibe (which reduces intestinal cholesterol absorption) and fibrates (targeting triglycerides) remain viable options, particularly in resource-limited settings. Ezetimibe, when combined with statins, has demonstrated incremental cardiovascular benefits in the IMPROVE-IT trial, albeit with more modest LDL-C reductions compared to PCSK9 inhibitors [5]. Fibrates, though less effective for LDL-C lowering, may be considered in patients with hypertriglyceridemia [7]. Future studies should explore the optimal combinations of these agents to balance efficacy, safety, and cost.

## 5. Limitations

Our study has several limitations inherent to a meta-analysis. While included studies were randomized trials, the associations of adverse events with degree of LDL-C lowering were observational. Given the lack of individual patient-level data, information regarding the type of muscle disorder, diabetes severity or control, and specific scores on neurocognitive tests is absent. In addition, there is heterogeneity in LDL-C levels in both the case and control groups. There is also a lack of individual patient data to assess dose–response relationships. There was an underrepresentation of certain populations (e.g., advanced CKD, extreme elderly) in some of the included studies. However, given the large number of patients in this analysis, our study sample provides an adequate sample size that individual studies do not have to identify a small excess risk of adverse events. Another important limitation is the relatively short follow-up duration of the PCSK9 inhibitor trials compared to the decades-long experience with statin therapy. Most included trials had a median follow-up of approximately 1.7 years, which may not fully capture the potential long-term adverse effects of profound LDL-C reduction. Although our findings align with existing pathophysiological evidence and prior trial results, extended observation periods are essential to definitively rule out rare or delayed adverse outcomes associated with extreme LDL-C lowering.

## 6. Future Directions

The ongoing VESALIUS-CV trial (NCT03872401) will provide five-year safety data on the effect of PCSK-9 inhibitors on cardiovascular outcomes in lower-risk patients without a history of MI or stroke [50]. Future studies should evaluate very low LDL-C in unique populations (e.g., heart failure, stroke), assess combination therapies (e.g., PCSK9 inhibitors + inclisiran), investigate non-cardiovascular effects (e.g., cancer, infection risk) with longer follow-ups, and incorporate patient-reported outcomes and quality-of-life measures.

## 7. Conclusions

Our analysis of data from multiple large, randomized studies is reassuring and does not demonstrate an excess risk of neurocognitive disorders, cancer, cataract, hepatobiliary disorders, muscle disorders, diabetes, and adverse events leading to drug discontinuation in patients treated with PCSK9 inhibitors on a background of statin therapy who achieve very low LDL-C levels. More importantly, the benefits of a reduction in MACEs continue to accrue in these patients very low LDL-C levels even below the currently recommended goals. However, more studies with longer follow-ups are necessary to address the gaps in knowledge regarding the safety of intensive lipid-lowering associated with PCSK-9 inhibitors particularly in the long term beyond 5–10 years.

## Figures and Tables

**Table 1:** Study characteristics of the included randomized controlled trials. * Age cutoff for patients who had familial hypercholesterolemia ≥35 years for men and 45 years for women.

Trial	Patient Characteristics	n		PCKS9i Tested	LDL-C Values in the Treatment Group	LDL-C Values in the Control Group	Follow up Duration (months)
		Very Low LDL-C	Control				
AMG 145 [16]	Age: ≥20 and ≤85 years Population: Japanese	202	202	Evolocumab	Mean 26 mg/dL	Mean 103 mg/dl	3
GLAGOV [17]	Age: ≥18 years Population: North America	484	484	Evolocumab	Mean 37 mg/dL	Mean 93 mg/d	19
FOURIER [18]	Age: ≥40 and ≤85 years Population: Multinational	2669	23,313	Evolocumab	<19 mg/dL	≥68 mg/dL	26.4
Robinson et al. [19]	Age: ≥18 years Population: Multinational	839	1894	Alirocumab	<25 mg/dL	LDL ≥25 mg/dL	26
SPIRE 1 and 2 [20]	Age: Men ≥ 50 years *, Women ≥ 60 years * Population: Multinational	6285	13,697	Bococizumab	≤25 mg/dL	≥70 mg/dL	SPIRE 1: 7 SPIRE 2: 12
ODYSSEY OUTCOMES [21]	Age: ≥18 years Population: Multinational	730	2152	Alirocumab	<15 mg/dL for safety Analyses <25 mg/dL for efficacy analyses	≥50 mg/dL	36

**Table 2:** Baseline clinical characteristics.

	AMG 145 [16]		GLAGOV [17]		FOURIER [18]		Robinson et al. [19]		SPIRE 1 and 2 [20]		ODYSSEY OUTCOMES [21]	
	Very Low LDL-C	Control	Very Low LDL-C	Control	Very Low LDL-C	Control	Very Low LDL-C	Control	Very Low LDL-C	Control	Very Low LDL-C	Control
**%**	50	50	50	50	17	83	25	75	50	50	50	50
**Age (years)**	61	62	60	60	63	61	62	59	63	63	58	58
**Female Gender**	39	40	28	28	28	22	25	43	29	30	20	33
**CAD**	11	15	-	-	-	-	77	61	-	-	-	-
**PAD**	14	12	-	-	14	14			-	-	3	5
**DM**	51	47	20	22	34	38	42	28	48	47	29	28
**HTN**	72	75	82	84	81	80	-	-	81	80	-	-
**Current smokers**	26	23	26	23	32	28	22	26	25	24	25	26
**Prior MI**	-	-	35	35	81	81	-	-	-	-	-	-
**Prior PCI**	-	-	39	39	-	-	-	-	-	-	15	18

Note. Values are presented as %, mean or median (interquartile range). CAD: coronary artery disease; DM: diabetes mellitus; HTN: hypertension; MI: myocardial infarction; PAD: peripheral artery disease; PCI: percutaneous coronary intervention.

## Supplementary Materials

The following supporting information can be downloaded at: https://www.mdpi.com/article/10.3390/jcm14134562/s1, Table S1. PRISMA 2020 Checklist Summary; Figure S1. Assessment of risk of bias risk for included studies with the Cochrane Collaboration tool; Figure S2: (A) Funnel plot for MACE; (B) Funnel plot for Neurocognitive disorder; (C) Funnel plot for Diabetes mellitus; (D) Funnel plot for Muscle disorders; (E) Funnel plot for any adverse events; (F) Funnel plot for Events leading to drug discontinuation; (G) Funnel plot for Cataract; (H) Funnel plot for Hepatobiliary disorders.

## Abbreviations

CI	Confidence interval
LDL-C	Low-density lipoprotein cholesterol
MACE	Major adverse cardiovascular event
OR	Odds ratio
PCSK9	Proprotein convertase subtilisin/kexin type 9
PRISMA	Preferred Reporting Items for Systematic Reviews and Meta-Analyses
RCT	Randomized controlled trial
