# Supplementary material for: Safety and Efficacy of Achieving Very Low LDL Cholesterol Concentrations with PCSK9 Inhibitors
Source: J Clin Med. 2025 Jun 27;14(13):4562. doi: 10.3390/jcm14134562 (PMC12249667; doi:10.3390/jcm14134562)
Supplement: Supplementary file 1 [file jcm-14-04562-s001.zip › jcm-3725398-supplementary.pdf]

Supplementary Table S1. PRISMA 2020 Checklist Summary

PRISMA 2020 Checklist Summary

Section/Topic	Item #	PRISMA 2020 Requirement	Reported on Page #	Documentation Status
TITLE	1	Identifies as systematic review	Title page	✓ Fully reported
ABSTRACT	2	Structured summary	Abstract	✓ Fully reported
INTRODUCTION	3	Rationale	2	✓ Fully reported
	4	Objectives	2	✓ Fully reported
METHODS	5	Eligibility criteria	2	✓ Fully reported
	6	Information sources	2	✓ Fully reported
	7	Search strategy	Supplement	✓ Fully reported
	8	Selection process	2	✓ Fully reported
	9	Data collection process	2	✓ Fully reported
	10a	Outcomes	5	✓ Fully reported
	10b	Other variables	Tables 1-2	✓ Fully reported
	11	Risk of bias assessment	Supplement	✓ Fully reported
	12	Effect measures	5	✓ Fully reported
	13a-f	Synthesis methods	5	✓ Fully reported
	14	Reporting bias assessment	5	✓ Fully reported
	15	Certainty assessment	-	✓ Fully reported
RESULTS	16a	Study selection	6 (Fig 1)	✓ Fully reported
	16b	Excluded studies	Supplement	✓ Fully reported
	17	Study characteristics	7 (Table 1)	✓ Fully reported
	18	Risk of bias in studies	Supplement	✓ Fully reported
	19	Results of individual studies	7	✓ Fully reported
	20a	Synthesis characteristics	8	✓ Fully reported
	20b	Synthesis results	8	✓ Fully reported

	20c	Heterogeneity	8	✓ Fully reported
	20d	Sensitivity analyses	-	✓ Fully reported
	21	Reporting biases	8	✓ Fully reported
	22	Certainty of evidence	-	◐ Partially reported
DISCUSSION	23a	Interpretation	8-10	✓ Fully reported
	23b	Limitations	10	✓ Fully reported
	23c	Review limitations	10	✓ Fully reported
	23d	Implications	10	✓ Fully reported
OTHER INFO	24a	Registration	-	○ Not reported
	24b	Protocol	-	○ Not reported
	24c	Protocol amendments	-	○ Not applicable
	25	Funding	Title page	✓ Fully reported
	26	Competing interests	Title page	✓ Fully reported
	27	Availability of data	-	✓ Fully reported

Supplementary Figure S1. Assessment of risk of bias risk for included studies with the Cochrane Collaboration tool.

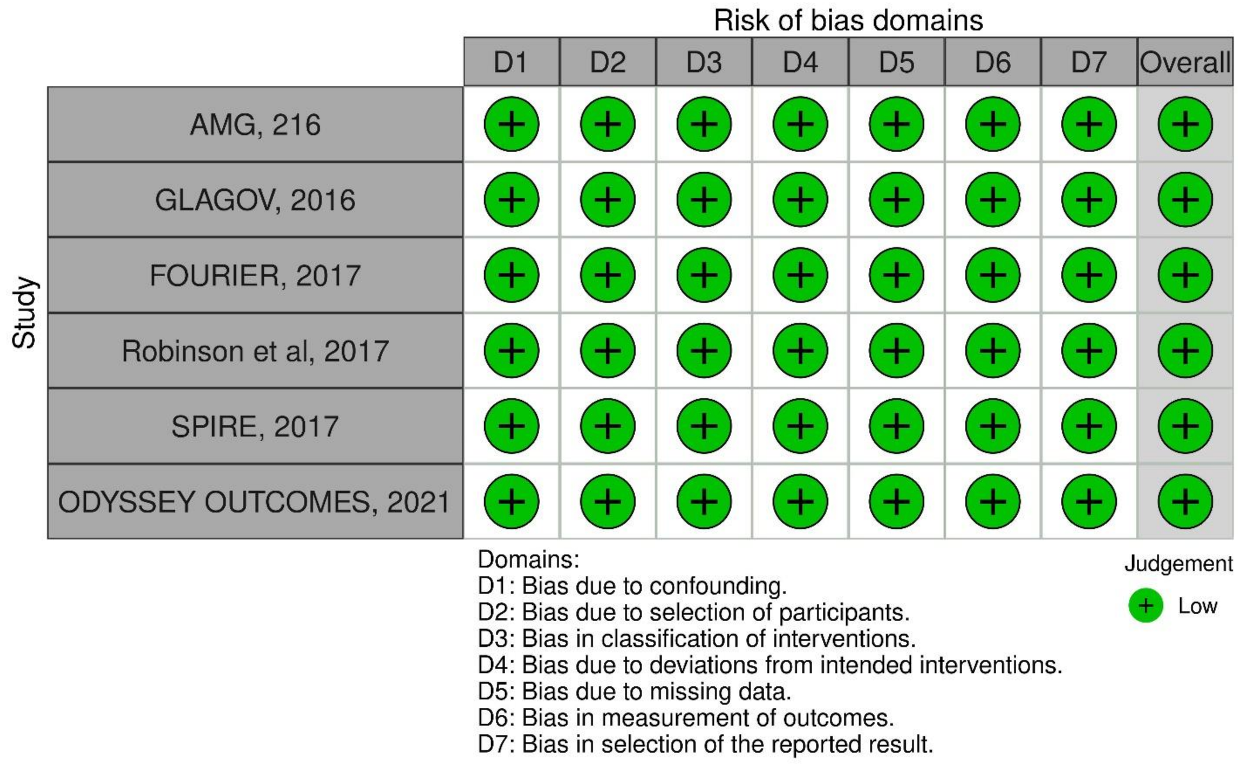

Supplementary Figure S2A. Funnel plot for MACE

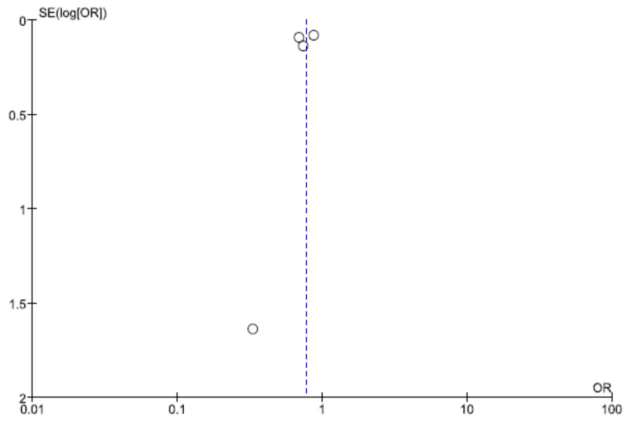

Supplementary Figure S2B. Funnel plot for Neurocognitive disorder

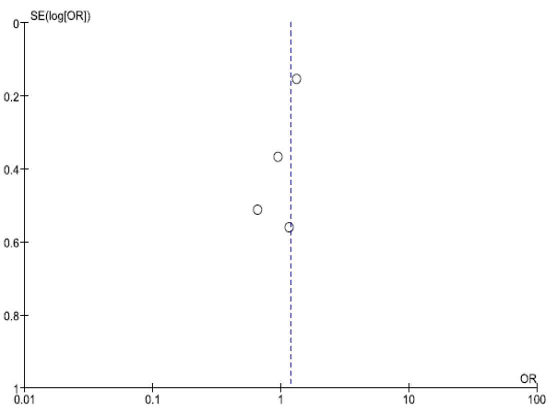

Supplementary Figure S2C. Funnel plot for Diabetes mellitus

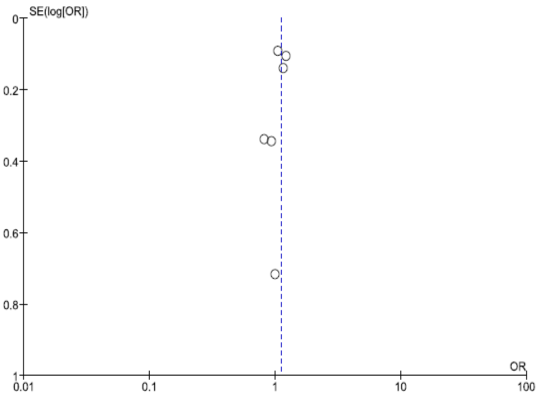

Supplementary Figure S2D. Funnel plot for Muscle disorders

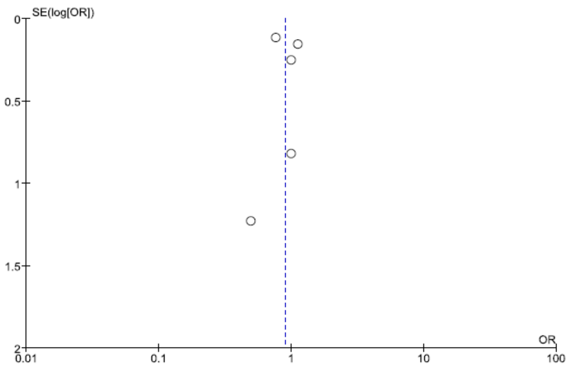

Supplementary Figure S2E. Funnel plot for any adverse events

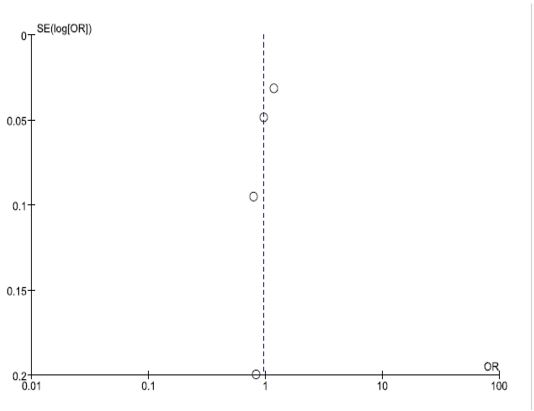

Supplementary Figure S2F. Funnel plot for Events leading to drug discontinuation

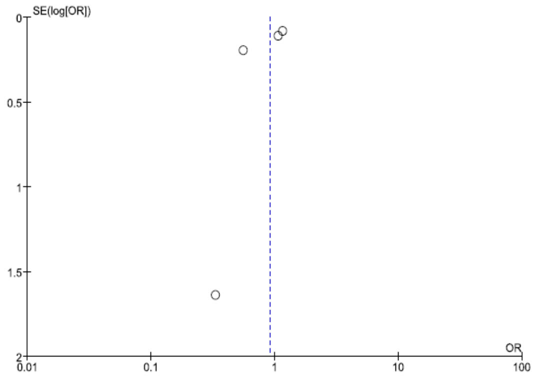

Supplementary Figure S2G. Funnel plot for Cataract

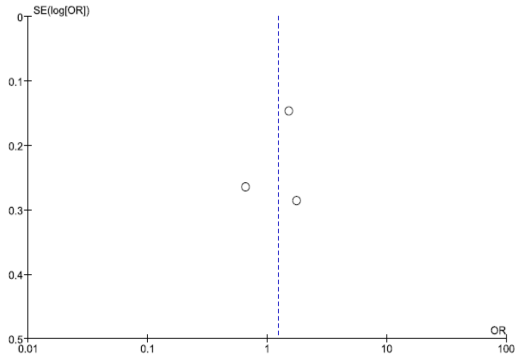

Supplementary Figure S2H. Funnel plot for Hepatobiliary disorders

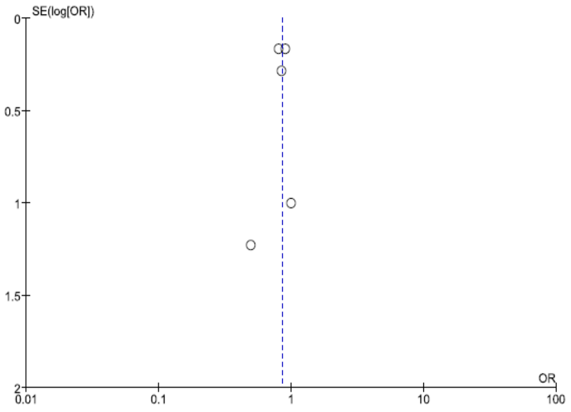
SE(log[OR])
0
0.5
1
1.5
2
OR
0.01
0.1
1
10
100
